# Molecular and Cellular Mechanisms of Epilepsy

**DOI:** 10.3390/ijms241512415

**Published:** 2023-08-04

**Authors:** Aleksey V. Zaitsev, Roustem Khazipov

**Affiliations:** 1 Sechenov Institute of Evolutionary Physiology and Biochemistry, 194223 Saint Petersburg, Russia; 2 Laboratory of Neurobiology, Kazan Federal University, 420008 Kazan, Russia; roustem.khazipov@inserm.fr; 3 Institut de Neurobiologie de la Méditerranée (Inserm U1249), Aix-Marseille Université, 13273 Marseille, France

Despite the availability of a large number of antiepileptic drugs, about 30% of patients with epilepsy, especially temporal lobe epilepsy (TLE), continue to experience seizures. The most rational therapeutic option for drug-resistant epilepsy is the prevention of the development and progression of epilepsy, based on an understanding of the pathophysiological mechanisms of epileptogenesis. Yet, current knowledge on the epileptogenic mechanisms is still insufficient. In recent years, many breakthroughs have been made in identifying molecular, cellular and network alterations associated with severe epilepsy. These alterations include but are not limited to (1) loss of principal cells and interneurons and neurogenesis, including changes in morphology and neuronal firing patterns related to altered composition or expression of receptors and channels; (2) gliosis, including changes in glial cell function and neuron–astrocyte interactions; (3) loss of blood–brain barrier integrity and neuroinflammation. All these histopathological changes are suspected to contribute to epileptogenesis and could constitute important targets for preventive therapies.

This Special Issue, “Molecular and Cellular Mechanisms of Epilepsy”, contains a selection of seven research papers and four reviews covering different aspects of the molecular and cellular biology of epilepsy. Two research papers analyze the role of glucocorticoids in patients with epilepsy. The glucocorticoid receptor (GR) has already been shown to be an important player in drug-resistant epilepsy due to focal cortical dysplasia (FCD), but the individual roles and clinical significance of the brain GRα and GRβ isoforms in FCD have not been well defined. The study by Rosemary Westcott et al. demonstrates for the first time that upregulated GRβ or a decreased GRα/GRβ ratio in the dysplastic brain may contribute to the pathogenesis and drug response in pharmacoresistant epilepsy, particularly in certain subgroups of patients such as women and those over 45 years of age [1]. The article by Tatyana Druzhkova et al. provides new evidence that the hypothalamic–pituitary–adrenal (HPA) axis, inflammatory processes, and neurotrophic factor systems are involved in the pathogenesis of epilepsy. As one of the hypotheses, the authors of the study propose that epilepsy is a model of chronic stress for human beings. This hypothesis is supported by the fact that patients with focal epilepsy have higher levels of cortisol in their blood serum than healthy people [2]. In addition, patients with epilepsy have a high incidence of comorbid depressive disorders, one of the causes of which is stress.

Epilepsy is a risk factor not only for depressive disorders but also for many other neurological diseases. For example, there is a reciprocal relationship between epilepsy and Alzheimer’s disease (AD). Epilepsy is a risk factor for AD, and AD is an independent risk factor for developing epilepsy in old age. An insightful article by Miren Altuna et al. analyzes common pathophysiological processes in both AD and epilepsy, including neuronal hyperexcitability and early excitatory–inhibitory dysregulation leading to dysfunction in the inhibitory GABAergic and excitatory glutamatergic systems [3].

Although not fully understood, the aberrant regulation of gene expression, including post-transcriptional networks, may be involved in the pathological mechanism underlying TLE. An abnormal expression of non-coding RNA is observed in patients with epilepsy and in animal models of epilepsy. The role of non-coding RNAs, such as microRNAs, long non-coding RNAs and circular RNAs, in the pathophysiology of epilepsy and their use as potential biomarkers for future therapeutic approaches is discussed in the review by Ida Manna et al. [4].

Animal models remain one of the most important tools in the search for the effective and safe treatment of pharmacoresistant epilepsy. Transplantation of γ-aminobutyric acid (GABA)-releasing cells could be used to counteract the imbalance between excitation and inhibition within epileptic neuronal networks and represent a novel treatment strategy. Eliška Waloschková et al. provide proof of concept that human embryonic stem-cell-derived GABAergic neurons can exert a therapeutic effect in epileptic animals, presumably by forming inhibitory synapses with host neurons [5]. Elena Proskurina et al. used an optogenetic approach to test the efficacy of suppressing ictal activity in entorhinal cortex slices of rodents in a 4-aminopyridine model using a low-frequency light stimulation of different target neurons. The main conclusion of this study was that a less specific and more generalized optogenetic stimulation of entorhinal cortex neurons was more effective in suppressing ictal activity in this model [6].

The study by Anna Volnova et al. addresses the important question of the role of astrocytes in epileptic activity by examining the effect of the gap junction blocker carbenoxolone (CBX) on epileptic activity in vitro and in vivo. Although the full mechanism of the suppression of epileptic activity by CBX is still unclear, it is highly likely that the astrocytic syncytium plays a major role in this mechanism [7]. The dysfunction of astrocytes in epilepsy may affect synaptic plasticity as well. The work of Postnikova et al. using the lithium–pilocarpine model in three-week-old rats shows a decrease in long-term potentiation, which the authors explain by a disruption in the interaction between neurons and astrocytes [8].

Abnormal epileptic activity in the brain causes significant metabolic and signaling imbalances, and the study by Lev Zavileyskiy et al. unravels the involvement of SIRT5 and OGDH in metabolic adaptation to single and chronic pentylenetetrazole-induced seizures through protein acylation [9]. The authors suggest that targeting post-translational acylation may be a general strategy for the treatment of epilepsy and other neurological disorders.

Two excellent reviews by Allan Kalueff’s group provide translational insights into epilepsy from zebrafish models [10,11]. One review discusses recent advances and current challenges in the development of experimental models of mTOR-dependent epilepsy and other related mTORopathies [10]. The other review focuses on current perspectives and challenges in the development of genetic, pharmacological and other experimental models of major CNS channelopathies based on zebrafish [11].

In conclusion, this Special Issue highlights the diversity of approaches used to study the molecular and cellular mechanisms of epilepsy, ranging from the development of new animal models to the use of non-coding RNAs, human embryonic stem cells, optogenetics, and so on. Continuing to elucidate the underlying molecular mechanisms of epilepsy and epileptogenesis and integrating novel drug, cell and gene therapy strategies into controlled clinical trials will allow for significant progress in a field where there are currently many unmet needs.
